# Two Cases of Mycobacterium shinjukuense Pulmonary Disease With a Long-Term Response to Treatment With Clarithromycin, Rifampicin, and Ethambutol

**DOI:** 10.7759/cureus.52888

**Published:** 2024-01-24

**Authors:** Takeshi Imakura, Soji Kakiuchi, Hitomi Kagawa, Naoya Murakami, Takashi Haku

**Affiliations:** 1 Department of Respiratory Medicine, Tokushima Prefectural Central Hospital, Tokushima, JPN

**Keywords:** isoniazid, tuberculosis, clarithromycin, nontuberculous mycobacteriosis, mycobacterium shinjukuense

## Abstract

*Mycobacterium shinjukuense* is a nontuberculous mycobacterium, and standard treatment for the infection has not been established. We report two cases of *M. shinjukuense* pulmonary disease in which two patients were treated with clarithromycin (CAM), rifampicin (RFP), and ethambutol (EB). Based on computed tomography (CT) findings, the patients experienced improvement with treatment. Reports of multiple cases of *M. shinjukuense* pulmonary disease treated with clarithromycin, rifampicin, and ethambutol are valuable, and they suggest that this regimen may be a new treatment option.

## Introduction

*Mycobacterium shinjukuense* is a nontuberculous mycobacterium characterized by 16S ribosomal RNA (rRNA), *rpo* B, and *hsp*65 gene sequencing [1]. Its Runyon classification is group III, similar to the *Mycobacterium avium-intracellulare* complex (MAC) [1]. However, although *M. shinjukuense* is a nontuberculous mycobacterium, rapid diagnostic methods for *Mycobacterium tuberculosis* (TB), including the TRC Rapid M.TB assay (Tosoh, Tokyo, Japan) and the DNA probe FR-MTD (Fujirebio Inc., Tokyo, Japan), can yield false-positive results [2-5]. Such results occur because the nucleotide sequence of the 16S rRNA gene in *M. shinjukuense* is highly homologous to its counterpart in the TB complex (97.8% homology with the TB ATCC 27294 strain) [1].

*Mycobacterium shinjukuense *was first isolated in 2004 in the Shinjuku ward, a central location in Tokyo, Japan, and was first reported in 2011 by Saito et al. [1]. To date, 23 cases of* M. shinjukuense* infection have been reported in Japan and Korea [1-11]. However, its pathogenicity and prognosis are unknown, and there is no established treatment. The aim of reporting two cases of* M. shinjukuense* pulmonary disease is to help fill that knowledge gap.

## Case presentation

Case 1

A 74-year-old female visited her primary care doctor because of a cough and bloody sputum at the end of June 2018. She had a history of osteoporosis but no history of respiratory diseases or smoking and no episodes of suspected exposure to water or soil. She was prescribed a cough suppressant, levofloxacin, and carbazochrome sodium sulfonate, and her symptoms improved after approximately two weeks. However, a chest radiograph obtained after her symptoms improved showed an enhanced infiltrative shadow in the right lower lung field. Therefore, the patient was referred to our hospital in early July 2018.

**Figure 1:**
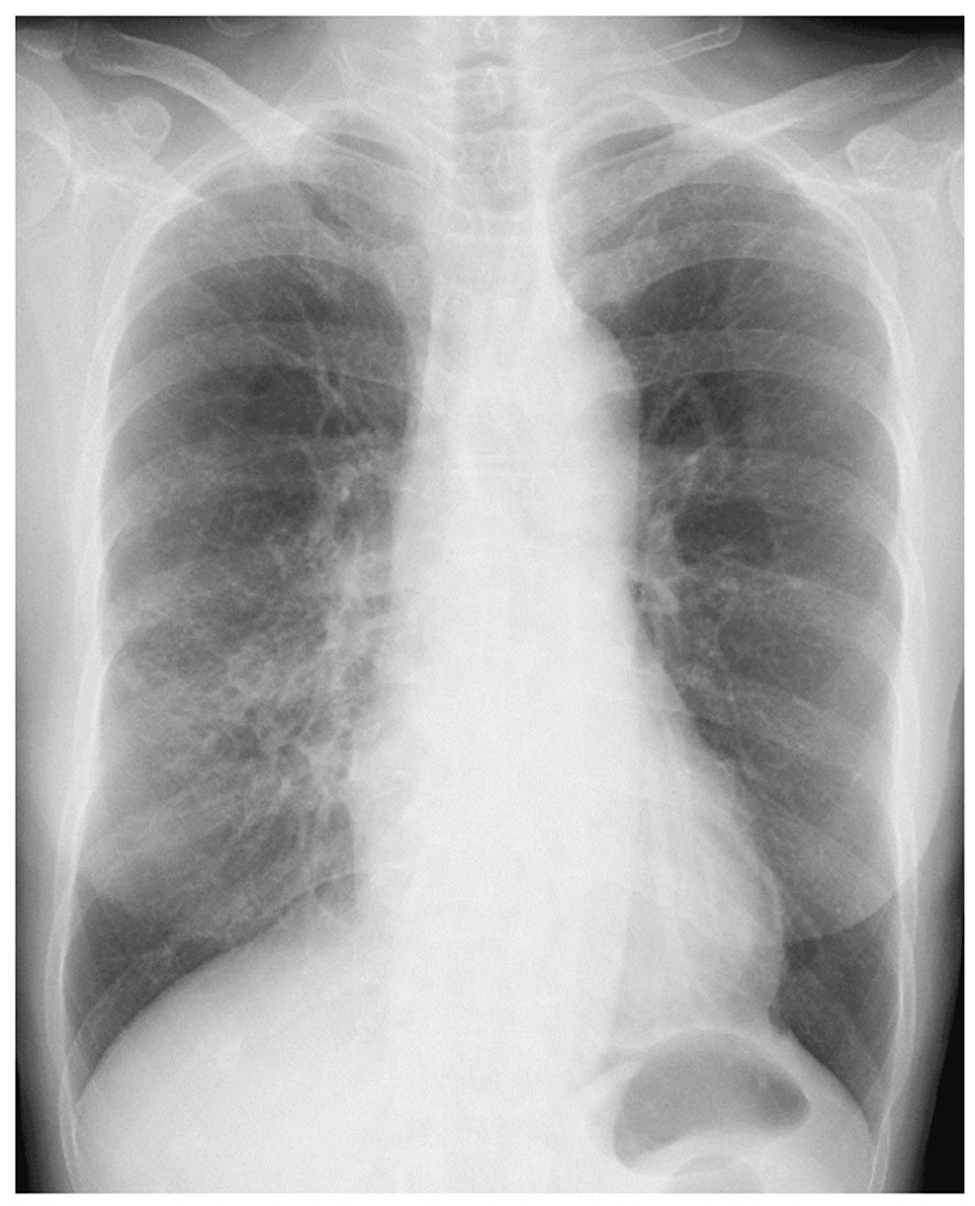
Chest radiography at the initial visit to our hospital of Case 1. Chest radiography at the initial visit to our hospital showed an infiltrative shadow with an air bronchogram and bronchiectasis in the right middle and lower lung fields.

**Figure 2:**
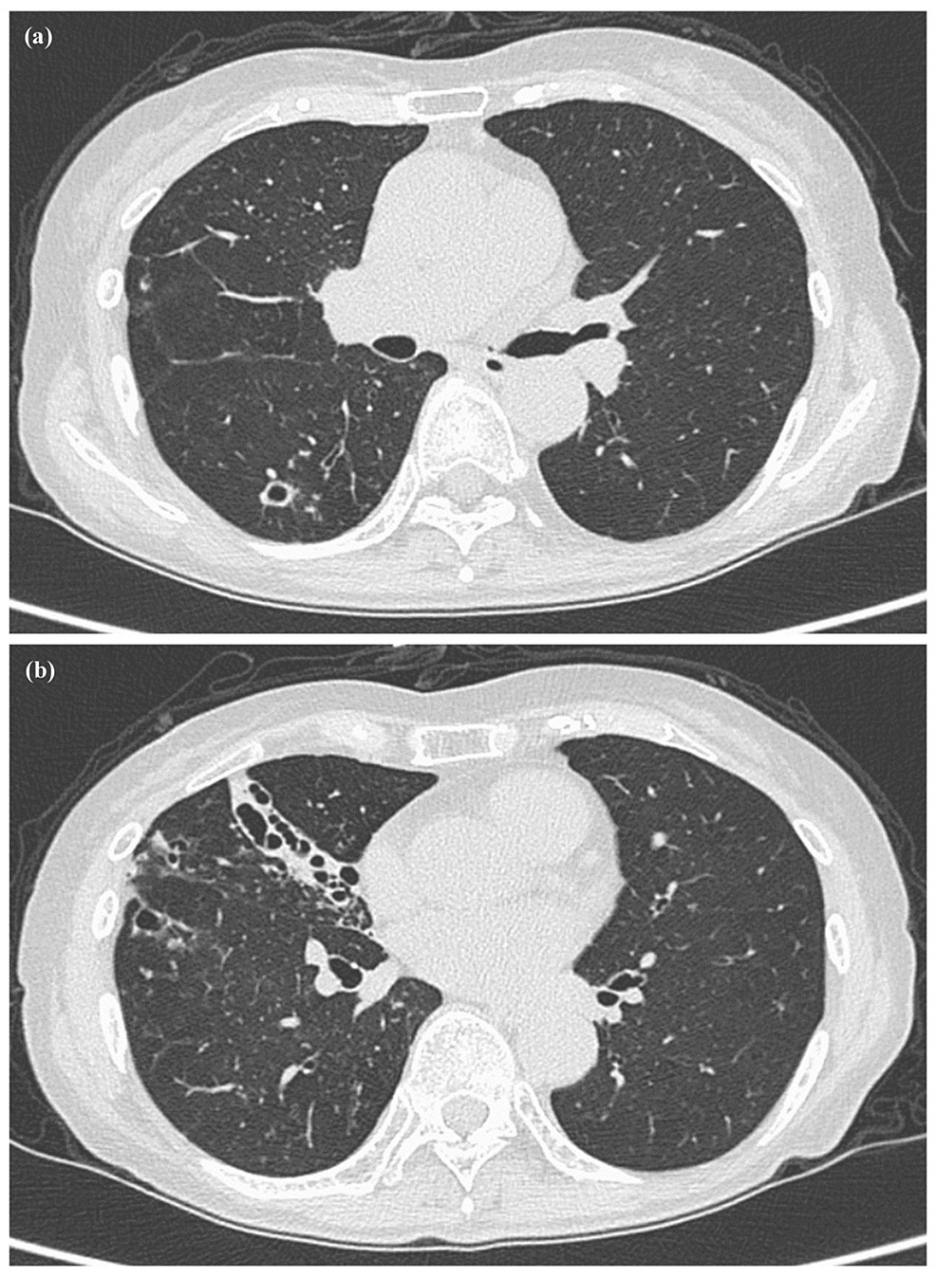
Chest computed tomography at the initial visit to our hospital of Case 1. Chest computed tomography in early July 2018 (at the initial visit) to our hospital showed a cavity in the right S6 region (a) and bronchiectasis mainly in the right middle lobe (b).

At the time of the first visit, her body mass index (BMI) was 19.8, and no abnormalities were observed in her vital signs or physical findings. Blood tests showed no abnormalities in the blood counts or biochemical tests. T-SPOT.TB was positive, but anti-MAC antibody was negative (Table 1). Sputum smear microscopy for acid-fast bacilli, which was performed at the initial visit, was negative. The results of the sputum polymerase chain reaction (PCR) for TB (COBAS TaqMan MTB, Roche, Basel, Switzerland) and MAC (COBAS TaqMan 48, Roche, Basel, Switzerland) were negative (Table 2). Chest radiography revealed an infiltrating shadow with an air bronchogram and bronchiectasis in the right middle and lower lung fields (Figure 1). Chest computed tomography (CT) revealed bronchiectasis mainly in the right middle lobe and a cavity in the right S6 region (Figure 2).

**Table 1:** The serum test results at the initial visit to our department of Case 1. WBC, white blood cell; Neut, neutrophil; Eo, eosinophil; Baso, basophil; Mono, monocyte; Lymp, lymphocyte; RBC, red blood cell; Hb, hemoglobin; Plt, platelet; AST, aspartate aminotransferase; ALT, alanine aminotransferase; ALP, alkaline phosphatase; LDH, lactate dehydrogenase; γ-GTP, gamma-glutamyl transpeptidase; BUN, blood urea nitrogen; Cre, creatinine; HbA1c, glycated hemoglobin; TP, total protein; Alb, albumin; CRP, C-reactive protein; KL-6: Krebs von den Lungen-6; CEA, carcinoembryonic antigen; CYFRA, cytokeratin 19 fragment; MAC, *Mycobacterium avium-intracellulare* complex

Parameters	Results	Reference range
WBC	6200/µL	3500-9100/µL
Neut	69.80%	
Eo	0.80%	
Baso	0.80%	
Mono	6.20%	
Lymp	22.40%	
RBC	4.07×10⁴/µL	3.76-5.00×10⁴/µL
Hb	12.5 g/dL	11.3-15.2 g/dL
Plt	21.1×10⁴/µL	13-36.9×10⁴/µL
AST	17 U/L	10-35 U/L
ALT	9 U/L	5-40 U/L
ALP	119 U/L	38-113 U/L
LDH	165 U/L	124-222 U/L
ɤ-GTP	19 U/L	0-30 U/L
BUN	12 mg/dL	7-20 mg/dL
Cre	0.68 mg/dL	0.4-0.9 mg/dL
Na	140 mEq/dL	135-146 mEq/dL
K	5.1 mEq/dL	3.5-4.8 mEq/dL
Glucose	88 mg/dL	60-110
HbA1c	5.40%	4.6%-6.2%
TP	6 g/dL	6.5-8.2 g/dL
Alb	4.4 g/dL	3.8-5.3 g/dL
KL-6	336 U/mL	50-500 U/mL
CRP	0 mg/dL	0-0.5 mg/dL
CEA	1.2 ng/mL	0-5 ng/mL
CYFRA	1 ng/mL	0-3.5 ng/mL
T-SPOT	(+)	
Panel A	16 spots	0-7 spots
Panel B	23 spots	0-7 spots
Anti-MAC antibody	(-)	
Aspergillus antigen	(-)	

**Table 2:** Acid-fast bacteriology test of Case 1. TB, *Mycobacterium tuberculosis*; MAC, *Mycobacterium avium-intracellulare* complex; PCR, polymerase chain reaction

Sputum collected in early July 2018	
Smear	(-)
TB-PCR	(-)
MAC-PCR	(-)
Culture	*Mycobacterium shinjukuense* (six-week incubation)
Bronchial lavage collected in late July 2018	
Smear	1+
TB-PCR	(-)
MAC-PCR	(-)
Culture	*Mycobacterium shinjukuense* (six-week incubation)

As nontuberculous mycobacterial lung disease was suspected on CT, bronchial lavage was performed in the right middle lobe in late July 2018. The smear microscopy of bronchial lavage fluid for acid-fast bacilli was 1+, PCR analyses of bronchial lavage for TB (COBAS TaqMan MTB) and MAC (COBAS TaqMan 48) were both negative. *Mycobacterium shinjukuense* was isolated from sputum collected at the initial examination and bronchial lavage fluid after six weeks of incubation by mass spectrometry (matrix-assisted laser desorption/ionization-time-of-flight mass spectrometry {MALDI-TOF MS}) (Table 2), resulting in the diagnosis of *M. shinjukuense* pulmonary disease.

**Figure 3:**
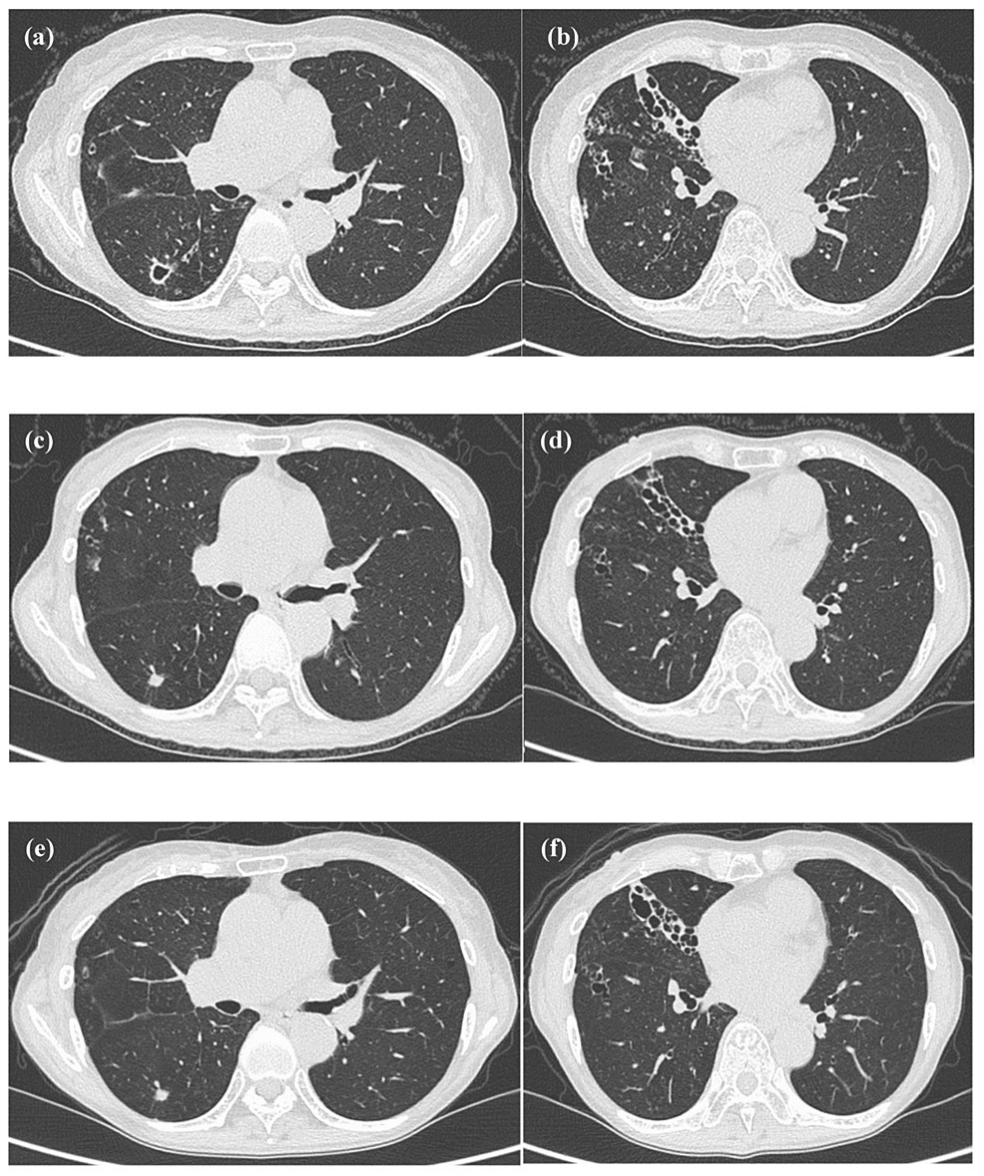
Progress of computed tomography of Case 1. (a and b) Computed tomography (CT) in mid-January 2019 showed an enlarged cavity and worsening infiltrative shadow. (c and d) CT in mid-January 2020 showed a reduction in the cavity and an improvement in the infiltrative shadow. (e and f) CT in mid-March 2022 showed no sign of the re-expansion of the cavity or the reaggravation of the infiltrative shadow.

The patient was followed up without treatment after the diagnosis. However, because chest CT showed worsening of the shadows (Figure 3a, 3b), treatment with clarithromycin (CAM), rifampicin (RFP), and ethambutol (EB) was initiated in mid-January 2019. After the initiation of treatment, the shadows on chest CT improved (Figure 3c, 3d), and the respiratory symptoms disappeared. However, treatment was terminated in mid-January 2020, as vision loss appeared with the administration of EB. The patient’s visual acuity improved after stopping treatment for *M. shinjukuense*. After the start of treatment, the amount of sputum decreased, and a sputum examination could not be performed. However, in July 2020, a sputum acid-fast bacilli culture was negative. The CT findings in March 2022 remained stable with no recurrence or progression (Figure 3e, 3f).

Case 2

An 82-year-old female visited her primary care physician in early August 2022 because of a fever. She had no specific medical history and no history of respiratory disease or smoking. She was a floriculturist and had a history of daily exposure to water and soil. She was diagnosed with COVID-19 infection using a COVID-19 antigen test and was referred to our department for close examination and treatment for hypoxemia.

**Figure 4:**
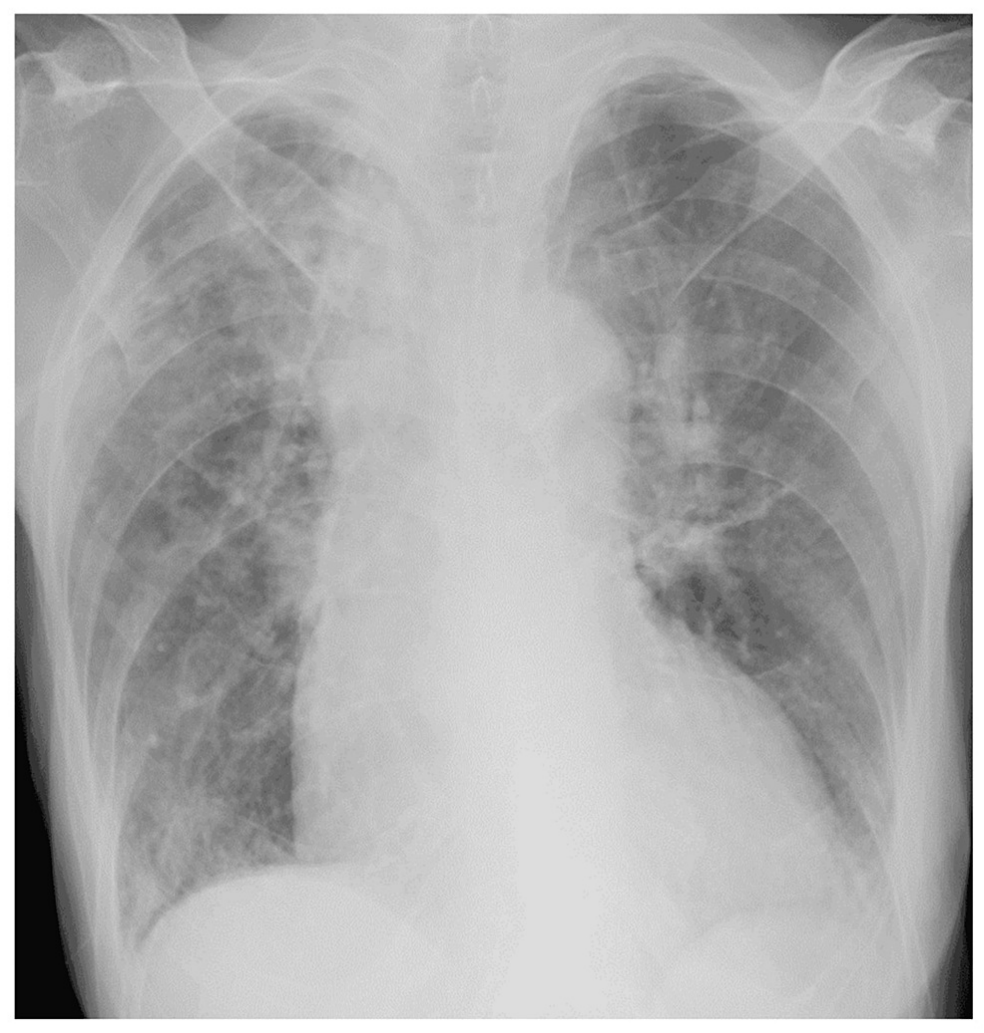
Chest radiography at the initial visit to our hospital of Case 2. Chest radiography at the initial visit to our hospital showed an infiltrating shadow in the right upper and lower lung fields.

**Figure 5:**
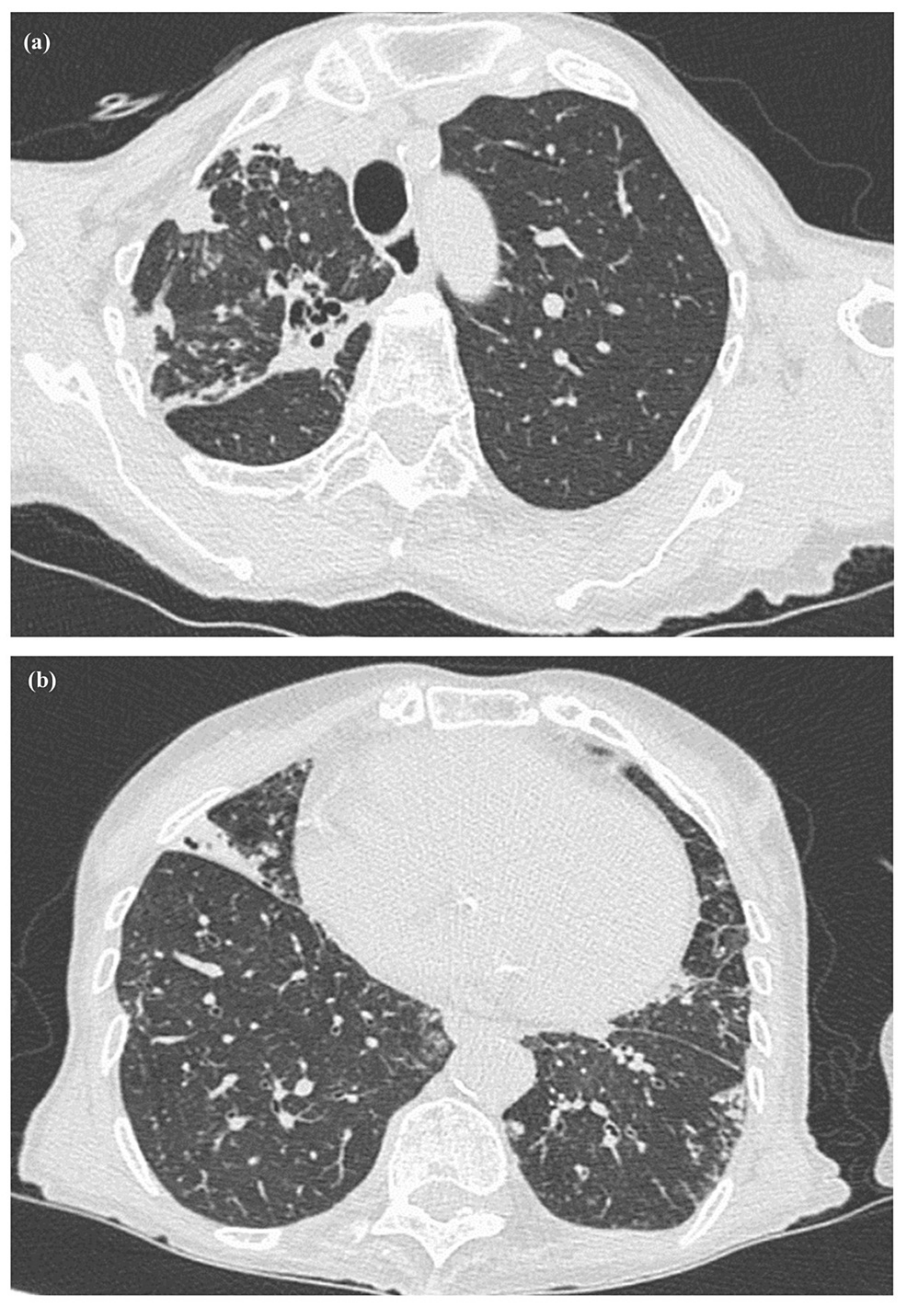
Computed tomography at the initial visit to our hospital of Case 2. Computed tomography (CT) showed an infiltrative shadow and bronchiectasis in the right upper (a) and middle lobes and lobular central granular shadows in both lower lobes (b).

The patient had a fever of 38°C and oxygen saturation of 86% in room air. Her BMI was 17.8, and there were no abnormalities in her physical findings at the time of the first visit. Blood tests showed no abnormalities in blood counts, but biochemical tests showed a mild increase in C-reactive protein. T-SPOT tests were negative for TB and anti-MAC antibodies (Table 3). Sputum smear microscopy for acid-fast bacilli, which was performed at the initial visit, was negative. PCR assays of sputum samples were negative for TB (COBAS TaqMan MTB) and MAC (COBAS TaqMan 48) (Table 4). Chest radiography revealed an infiltrating shadow in the right upper and lower lung fields (Figure 4). Chest CT showed an infiltrative shadow and bronchiectasis in the right upper and middle lobes and lobular central granular shadows in both lower lobes (Figure 5).

**Table 3:** The serum test results at the initial visit to our department of Case 2. WBC, white blood cell; Neut, neutrophil; Eo, eosinophil; Baso, basophil; Mono, monocyte; Lymp, lymphocyte; RBC, red blood cell; Hb, hemoglobin; Plt, platelet; AST, aspartate aminotransferase; ALT, alanine aminotransferase; ALP, alkaline phosphatase; LDH, lactate dehydrogenase; γ-GTP, gamma-glutamyl transpeptidase; BUN, blood urea nitrogen; Cre, creatinine; HbA1c, glycated hemoglobin; TP, total protein; Alb, albumin; CRP, C-reactive protein; KL-6, Krebs von den Lungen-6; MAC, *Mycobacterium avium-intracellulare* complex

Parameters	Results	Reference range
WBC	4500/µL	3500-9100/µL
Neut	55.20%	
Eo	0.20%	
Baso	0.20%	
Mono	6.90%	
Lymp	37.50%	
RBC	3.98×10⁴/µL	3.76-5.00×10⁴/µL
Hb	12.1 g/dL	11.3-15.2 g/dL
Plt	12.8×10⁴/µL	13-36.9×10⁴/µL
AST	29 U/L	10-35 U/L
ALT	10 U/L	5-40 U/L
ALP	39 U/L	38-113 U/L
LDH	218 U/L	124-222 U/L
ɤ-GTP	9 U/L	0-30 U/L
BUN	23.2 mg/dL	7-20 mg/dL
Cre	0.57 mg/dL	0.4-0.9 mg/dL
Na	139 mEq/dL	135-146 mEq/dL
K	4.6 mEq/dL	3.5-4.8 mEq/dL
Glucose	94 mg/dL	60-110
HbA1c	5.60%	4.6%-6.2%
TP	6.9 g/dL	6.5-8.2 g/dL
Alb	3.5 g/dL	3.8-5.3 g/dL
CRP	2.49 mg/dL	0-0.5 mg/dL
KL-6	518 U/mL	50-500 U/mL
T-SPOT	(-)	
Panel A	0 spots	0-7 spots
Panel B	0 spots	0-7 spots
Anti-MAC antibody	(-)	

**Table 4:** Acid-fast bacteriology test of Case 2. TB, *Mycobacterium tuberculosis*; MAC, *Mycobacterium avium-intracellulare* complex; PCR, polymerase chain reaction

Sputum collected in early August 2022	
Smear	(-)
TB-PCR	(-)
MAC-PCR	(-)
Culture	(-)
Bronchial lavage collected in late November 2022	
Smear	(-)
TB-PCR	(-)
MAC-PCR	(-)
Culture	*Mycobacterium shinjukuense* (eight-week incubation)

As nontuberculous mycobacterial lung disease was suspected on the basis of CT findings, bronchial lavage was performed in the right middle lobe in late November 2022. The smear microscopy of the bronchial lavage fluid for acid-fast bacilli was negative, and the PCR of the bronchial lavage for TB (COBAS TaqMan MTB) and MAC (COBAS TaqMan 48) was negative. *Mycobacterium shinjukuense* was isolated from bronchial lavage fluid after eight weeks of incubation by mass spectrometry (MALDI-TOF MS) (Table 4), which resulted in the diagnosis of *M. shinjukuense* pulmonary disease.

**Figure 6:**
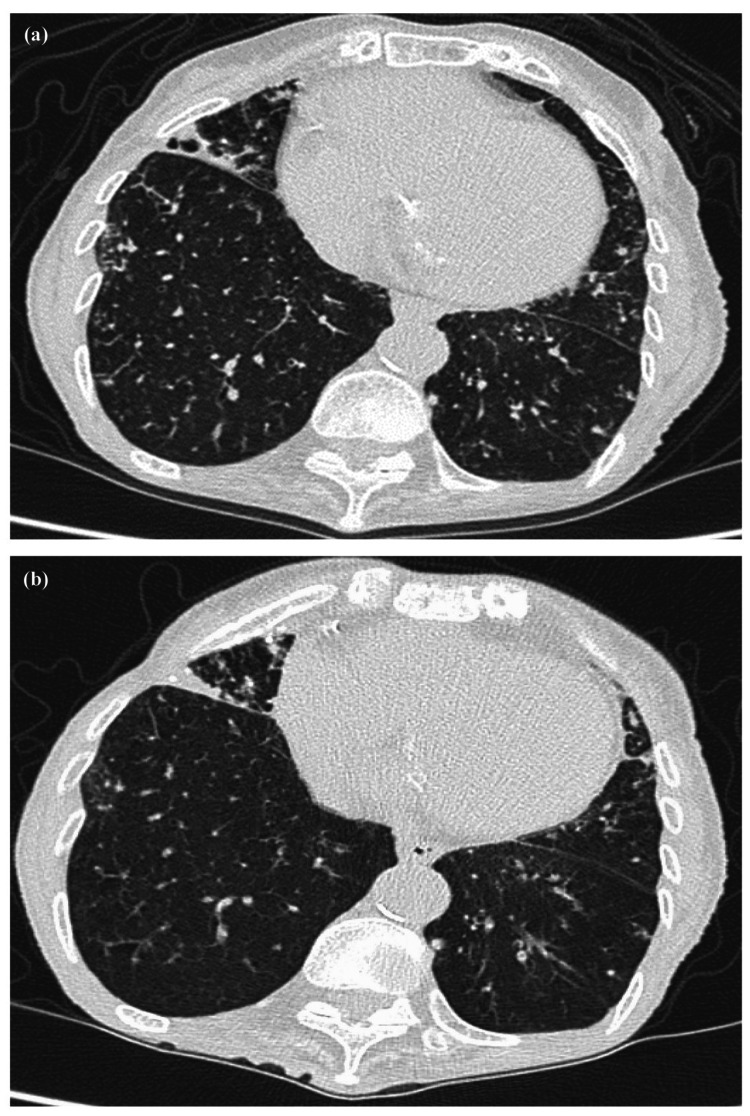
Progress of computed tomography of Case 2. (a) Computed tomography (CT) in mid-February 2023 showed no improvement of lobular central granular shadows in both lower lobes. (b) CT in early October 2023 showed the improvement of lobular central granular shadows in both lower lobes.

The patient was followed up without treatment for approximately six months after the diagnosis. However, because the structural destruction of the lungs had already progressed and CT showed no improvement in the shadows (Figure 6a), treatment with CMA, RFP, and EB was initiated in early February 2023. The lobular central granular shadows in both lower lobes have improved since the start of treatment (Figure 6b), and at the time of the writing of this report, treatment has been ongoing for 10 months and will continue in the future.

## Discussion

As *M. shinjukuense* is a relatively recently identified species, the evidence of its biology, etiology, and clinical behavior has not been sufficiently accumulated. Only 25 cases of *M. shinjukuense* pulmonary disease, including the current cases, were reported by December 2023 (Table 5) [1-11]. Among these cases, 24 were reported in Japan and one in Korea. The median age of the patients was 73 years (five males and 20 females). Among the 18 cases for which CT images were available, 10 showed nodules and bronchiectasis patterns, one showed a fibrocavitary pattern, and seven showed fibrocavitary plus nodules and bronchiectasis patterns. Although our two cases had no history of respiratory disease, five cases of* M. shinjukuense* pulmonary infection with a history of pulmonary TB have been reported, and it is possible that a history of respiratory disease is a risk factor for *M. shinjukuense *pulmonary infection. In addition, although we do not have enough data from previous reports, the BMI of our two cases was low. Therefore, it will be necessary to examine whether low BMI is a risk factor for *M. shinjukuense* pulmonary infections. One of our two cases had a history of concentrated exposure to water and soil, which may have been involved in *M. shinjukuense *pulmonary infection, and further case accumulation is needed. Compared with MAC pulmonary disease, no significant differences were observed with respect to age, sex ratio, or imaging findings [12].

**Table 5:** Case list and clinical features of M. shinjukuense pulmonary infection. RECAM, clarithromycin, rifampicin, and ethambutol; HRE, isoniazid, rifampicin, and ethambutol; EM, erythromycin; HREZ, isoniazid, rifampicin, ethambutol, and pyrazinamide; CAM, clarithromycin; HR, isoniazid and rifampicin; RCAM, clarithromycin and rifampicin; LVFX, levofloxacin; KM, kanamycin; M, male; F, female

Case	Country	Age	Sex	Drug regimens	Duration of treatment	Duration from the start of treatment to reporting	Outcome	Imaging finding	Underlying lung disease	Underlying disease	Reported year	Reference
1	Japan	78	M	Unknown	Unknown	Unknown	Unknown	Unknown	Unknown	Unknown	2011	Saito et al., 2011 [1]
2	Japan	89	M	Unknown	Unknown	Unknown	Unknown	Unknown	Unknown	Unknown	2011	Saito et al., 2011 [1]
3	Japan	57	F	Unknown	Unknown	Unknown	Unknown	Unknown	Unknown	Unknown	2011	Saito et al., 2011 [1]
4	Japan	64	F	Unknown	Unknown	Unknown	Unknown	Unknown	Unknown	Unknown	2011	Saito et al., 2011 [1]
5	Japan	73	F	Unknown	Unknown	Unknown	Unknown	Unknown	Unknown	Unknown	2011	Saito et al., 2011 [1]
6	Japan	73	F	Unknown	Unknown	Unknown	Unknown	Unknown	Unknown	Unknown	2011	Saito et al., 2011 [1]
7	Japan	89	F	Unknown	Unknown	Unknown	Unknown	Unknown	Unknown	Unknown	2011	Saito et al., 2011 [1]
8	Japan	64	F	RECAM	6	6	Improved	Nodules and bronchiectasis	None	None	2011	Futatsugi et al., 2011 [2]
9	Japan	80	F	HRE	1	1	Improved	Nodules and bronchiectasis	None	None	2013	Watanabe et al., 2013 [3]
10	Japan	73	F	HRE	5	5	Improved	Nodules and bronchiectasis	None	None	2015	Oshima et al., 2015 [6]
11	Korea	56	F	No treatment	(-)	(-)	(-)	Nodules and bronchiectasis	Old pulmonary tuberculosis	None	2015	Moon et al., 2015 [7]
12	Japan	57	F	EM	Unknown	Unknown	Improved	Nodules and bronchiectasis	None	Goiter	2016	Takeda et al., 2016 [4]
13	Japan	72	F	HRE	9	9	Improved	Nodules and bronchiectasis	Pulmonary *Mycobacterium avium*	Hypertension	2016	Takeda et al., 2016 [4]
14	Japan	83	F	HREZ→RECAM	Unknown	Unknown	Improved	Nodules and bronchiectasis	Pulmonary emphysema	Hypertension	2016	Takeda et al., 2016 [4]
15	Japan	75	M	HREZ→HRE	12	12	Improved	Fibrocavitary	Old pulmonary tuberculosis	Diabetes mellitus	2016	Takeda et al., 2016 [4]
16	Japan	82	M	HRE	Unknown	Unknown	Improved	Fibrocavitary+nodules and bronchiectasis	Old pulmonary tuberculosis	Prostate cancer	2016	Takeda et al., 2016 [4]
17	Japan	93	M	HRE→EM	Unknown	Unknown	No change	Fibrocavitary+nodules and bronchiectasis	Old pulmonary tuberculosis	Chronic heart failure	2016	Takeda et al., 2016 [4]
18	Japan	85	F	CAM→HRE	26	26	Improved	Fibrocavitary+nodules and bronchiectasis	Old pulmonary tuberculosis	None	2016	Hayashi et al., 2016 [8]
19	Japan	72	F	HR+CAM→RCAM+LVFX	18	18	Improved	Fibrocavitary+nodules and bronchiectasis	None	Polymyalgia rheumatica	2018	Meda et al., 2018 [9]
20	Japan	62	F	RECAM+KM→RECAM	30	30	Improved	Nodules and bronchiectasis	None	Hypertension	2018	Arai et al., 2018 [10]
21	Japan	68	F	RECAM＋KM→RECAM	3	3	Improved	Nodules and bronchiectasis	None	Hypertension and hyperlipidemia	2018	Arai et al., 2018 [10]
22	Japan	66	F	HRE	12	12	Improved	Fibrocavitary+nodules and bronchiectasis	None	None	2020	Taoka et al., 2020 [5]
23	Japan	59	F	RECAM	24	24	Improved	Fibrocavitary+nodules and bronchiectasis	None	Breast cancer	2023	Nakamura et al., 2023 [11]
24	Japan	74	F	RECAM	12	38	Improved	Fibrocavitary+nodules and bronchiectasis	None	Osteoporosis	2023	Present case
25	Japan	82	F	RECAM	10	10	Improved	Nodules and bronchiectasis	None	None	2023	Present case

*Mycobacterium shinjukuense* has been reported to have good susceptibility to isoniazid (INH), RFP, EB, and CAM in vitro [2-4,6]. However, in the case of nontuberculous mycobacteria, the results of the drug susceptibility test should be carefully interpreted, as the susceptibility in vitro and in vivo may not be consistent [13]. Although the standard chemotherapy for *M. shinjukuense* has not been established, in the past treated cases (excluding our cases), nine of 15 cases were treated with INH, RFP, and EB (including one case in which the initial regimen was INH+RFP+EB+pyrazinamide {PZA} and changed to CAM+RFP+EB during the course of treatment, one case that changed to erythromycin {EM} during the course of treatment, one case that switched from INH+RFP+EB+PZA, and one case that switched from CAM); five cases were treated with CAM+RFP+EB (including one case that switched from INH+RFP+EB+PZA treatment and two cases that switched from CAM+RFP+EB+kanamycin); and two cases were treated with EM (including one case that switched from INH+RFP+EB to EM and one case in which the initial regimen was INH+RFP+CAM and changed to RFP+CAM+levofloxacin) (Table 5) [2-11]. Many patients were treated with the anti-TB regimen, suggesting that they may have been diagnosed with pulmonary TB and started treatment due to a false-positive diagnosis based on rapid TB tests [3,4]. Based on previous reports, both treatment regimens were considered effective against *M. shinjukuense* (Table 5).

Although there have been many reports of *M. shinjukuense* treated with anti-TB drugs, as mentioned above, five cases of *M. shinjukuense* that responded to treatment with CAM, RFP, and EB have also been reported [2,4,10,11]. The current report adds two more. In Case 1 of our report, the patient completed 12 months of treatment and remained stable without treatment for 26 months. This case has the longest known treatment course for *M. shinjukuense* lung disease cases reported to date. In most of the reports to date, cases of *M. shinjukuense* treated with CAM, RFP, and EB have been reported in more detail than cases treated with anti-TB regimen. In many cases, as in Case 1 of the present case, the course of treatment is known for a relatively long time compared with cases treated with anti-TB drugs (Table 5).

The present case report has two limitations. First, we were unable to submit drug susceptibility tests for *M. shinjukuense* in either case. Although the in vitro drug sensitivity of nontuberculous mycobacteria is not necessarily reflective of in vivo conditions, we believe that it provides important information for determining treatment strategies. Second, nontuberculous mycobacteria may heal spontaneously without treatment, so it may not be possible to conclude that drug treatment was successful.

## Conclusions

We report two cases of *M. shinjukuense* that were treated with CAM, RFP, and EB regimens. Although the standard treatment for *M. shinjukuense* has not yet been established, our findings suggest that the combination of CAM, RFP, and EB could be an effective approach. Given the rarity of pulmonary infections caused by *M. shinjukuense*, further investigation through additional case studies is needed.
